# Supplementary material for: Superficial Slow Rhythms Integrate Cortical Processing in Humans
Source: Sci Rep. 2018 Feb 1;8:2055. doi: 10.1038/s41598-018-20662-0 (PMC5794750; doi:10.1038/s41598-018-20662-0)
Supplement: Supplementary file 1 — Supplementary Information [file 41598_2018_20662_MOESM1_ESM.pdf]

Title: Superficial Slow Rhythms Integrate Cortical Processing in Humans

Authors: Mila Halgren [a *], Daniel Fabo [b], István Ulbert [c,d], Joseph R. Madsen [e], Lorand Erőss [d,f], Werner K. Doyle[g], Orrin Devinsky[g], Donald Schomer[h], Sydney S. Cash[a †], Eric Halgren[i †]

Affiliations:

[a] Department of Neurology, Epilepsy Division, Massachusetts General Hospital, Harvard Medical School, Boston, MA 02114, USA.

[b] Epilepsy Centrum, National Institute of Clinical Neurosciences, Budapest, Hungary.

[c] Institute of Cognitive Neuroscience and Psychology, Research Center for Natural Sciences, Hungarian Academy of Science

[d] Péter Pázmány Catholic University, Faculty of Information Technology and Bionics, Budapest, Hungary.

[e] Departments of Neurosurgery, Boston Children's Hospital and Harvard Medical School, Boston, MA 02115, USA.

[f] Department of Functional Neurosurgery, National Institute of Clinical Neurosciences, Budapest, Hungary.

[g] Comprehensive Epilepsy Center, New York University School of Medicine, New York, NY 10016, USA.

[h] Department of Neurology, Beth Israel Deaconess Medical Center, Boston, MA 02215, USA

[i] Departments of Neurosciences and Radiology, Center for Human Brain Activity Mapping, University of California at San Diego, La Jolla, CA 92093, USA.

[*] Corresponding author

[†] Both authors contributed equally to this work.

The corresponding author is Mila Halgren - Department of Neurology, Epilepsy Division, Massachusetts General Hospital, Harvard Medical School, Boston, MA 02114, USA. (mhalgren@mit.edu)

Supplementary Figures and Captions

Supplementary Fig. S1

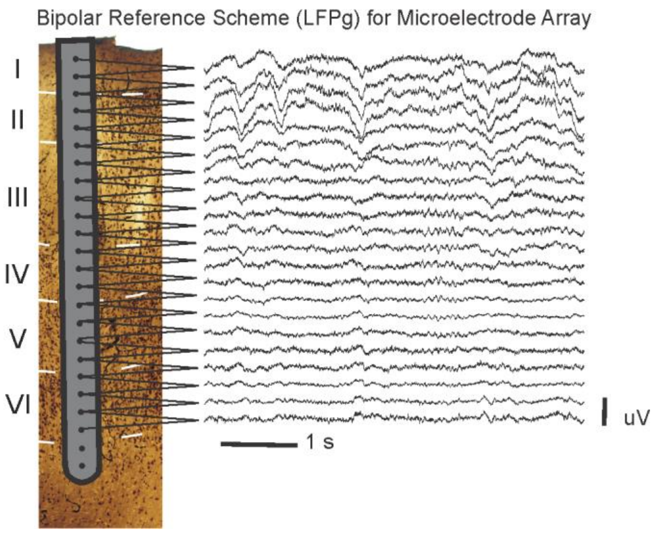

Reference scheme and representative data from a laminar microelectrode array. The local field potential gradient (LFPg) is the first spatial derivative of the LFP, with each channel referenced to its neighbor (directly below).

Supplementary Fig. S2

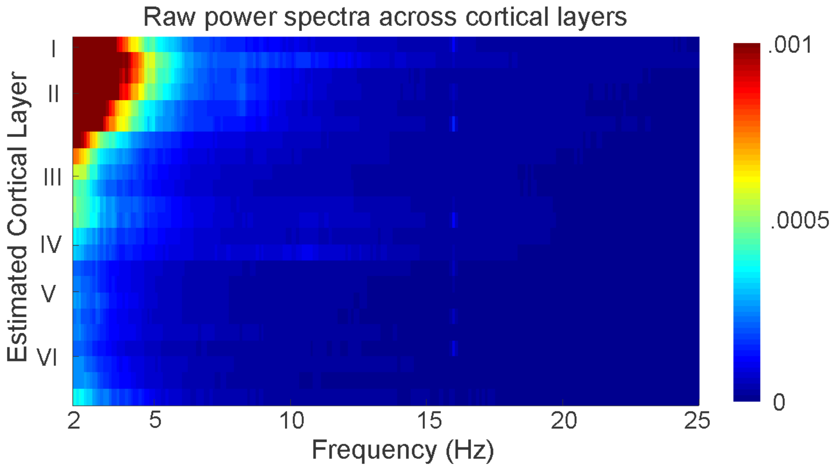

Grand averaged raw spectral power across cortical layers. Before averaging, power was normalized within subjects by dividing by the total amount of power from 1-40 Hz across all channels.

Supplementary Fig. S3

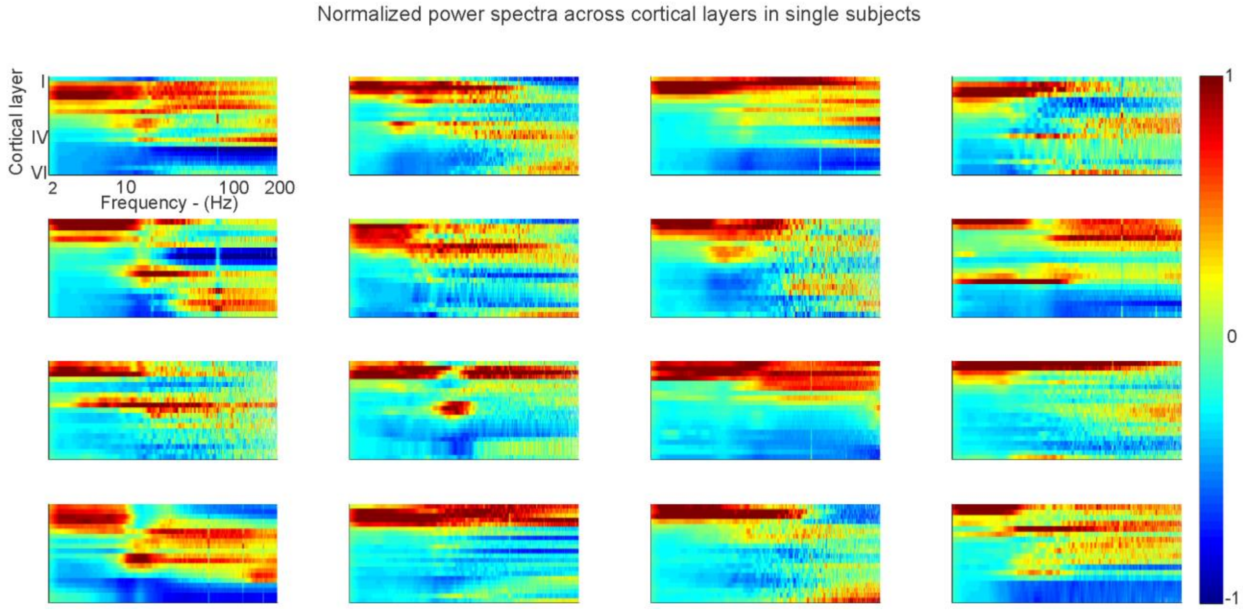

Delta/theta rhythms are generated in superficial cortical layers in 16 different regions/subjects. Single participant plots of the power spectral density z-normalized within channels/columns. Note that sub-10 Hz power is generated in superficial cortex across all participants. Note also that subjects 2, 3, 7, 8, 9 and 16 had no interpolated channels.

Supplementary Fig. S4

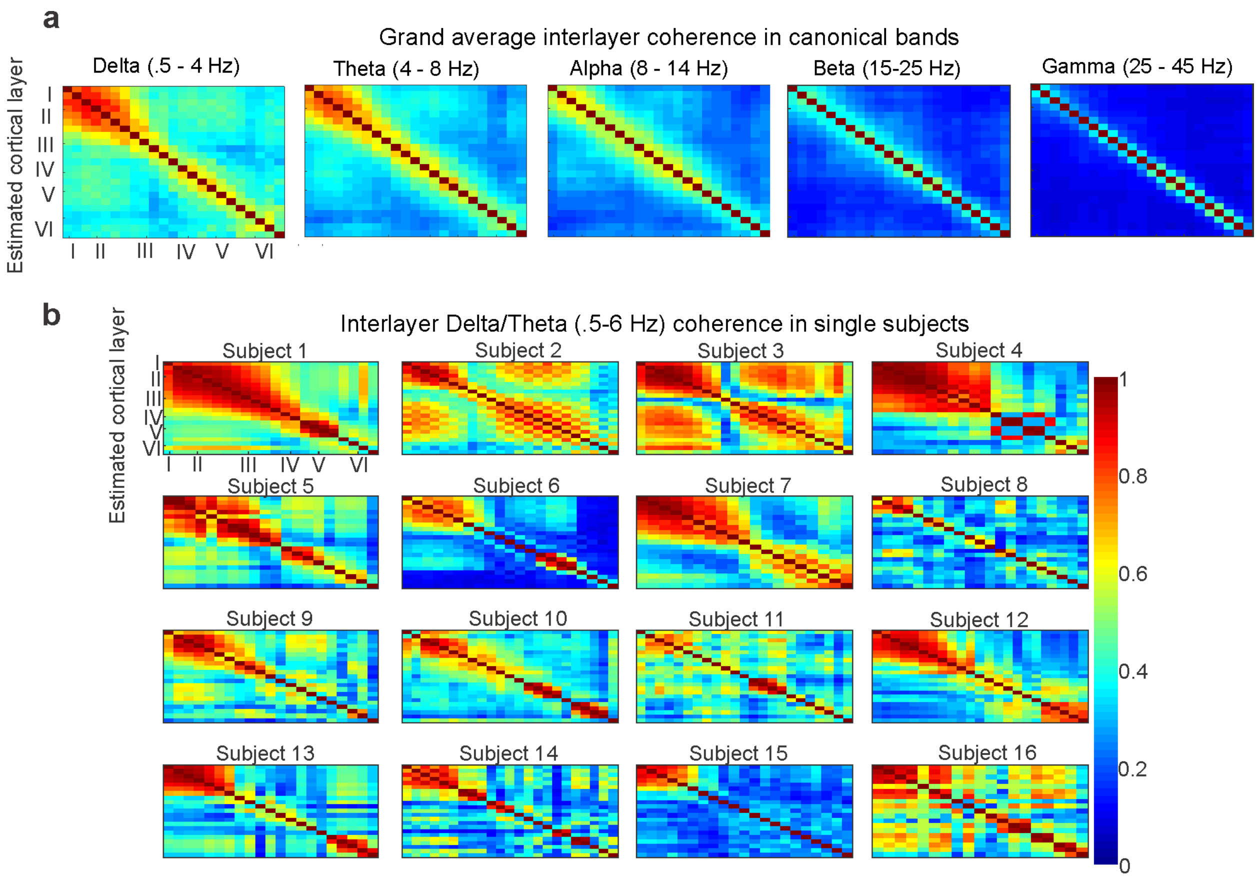

Slow rhythms are coherent within superficial cortex. (**a**) Grand averaged (n=16) interlayer coherence in canonical bands. Note that coherence is maximal in the delta band and superficial layers. (**b**) Single participant interlayer coherence in the delta/theta band. Slow coherence is maximal within superficial contacts across all recordings.

Supplementary Fig. S5

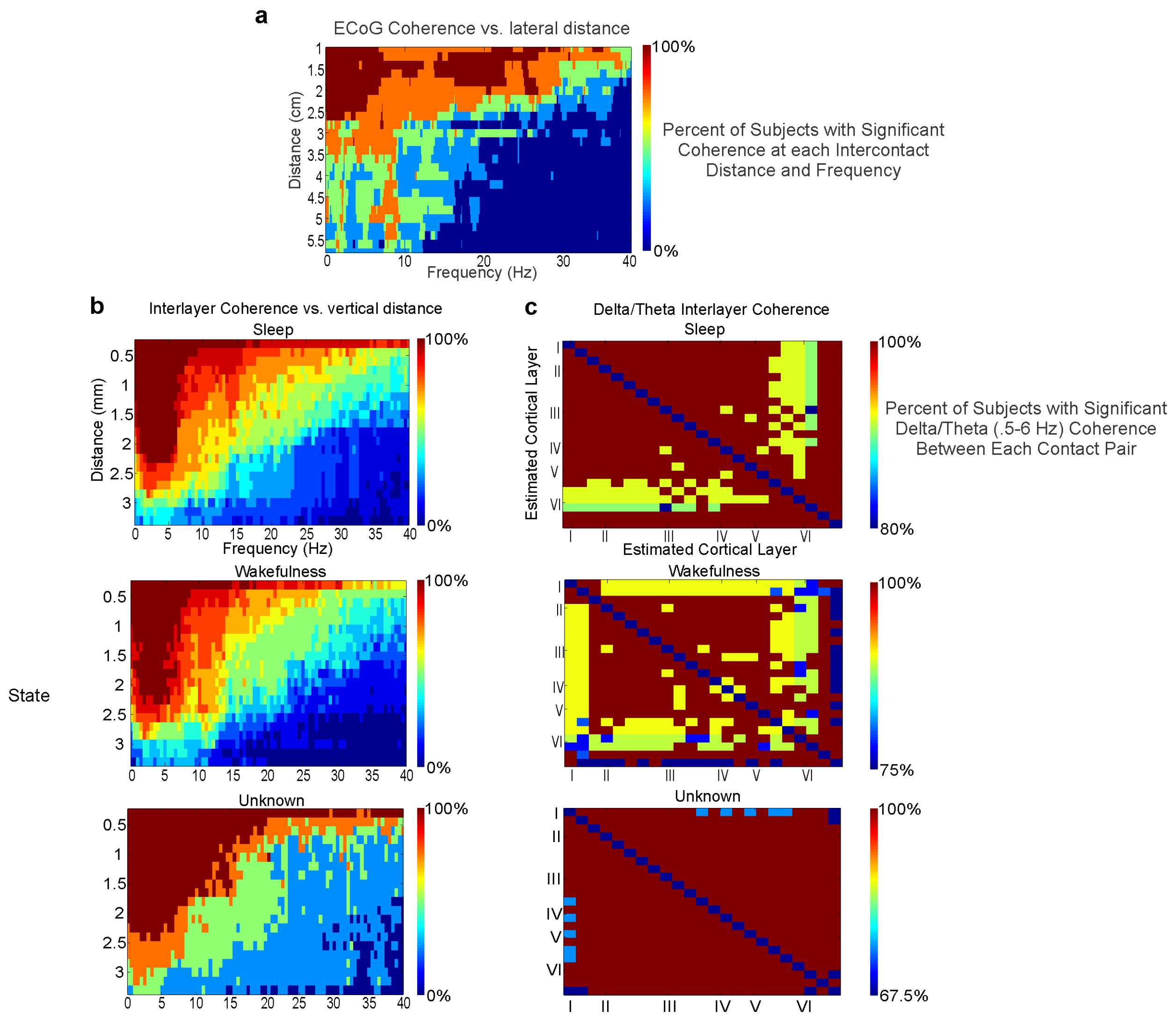

Number of subjects with significant coherence as measured by within-subjects non-parametric trial shuffling. (**a**) Percent of subjects (n=4) with significant coherence between ECoG contacts for each intercontact distance and frequency. The proportion of subjects with significant coherence decreases at high frequencies and distances. (**b**) Proportion of subjects with significant coherence between laminar contacts at each interlayer distance and frequency. A pattern of consistent coherence at short distances and low frequencies, very similar to lateral coherence, is observed. To verify that this held within different behavioral states, we assessed coherence

separately within wakefulness (n=10) and sleep (n=12). ‘Unknown’ refers to periods in which the patient’s state was not noted. (**c**) The percentage of subjects with significant delta/theta (.5-6 Hz) coherence for each contact pair within a single laminar array. Note that delta/theta is almost always coherent throughout the laminar depth, particularly in superficial cortex. cortex.

Supplementary Fig. S6

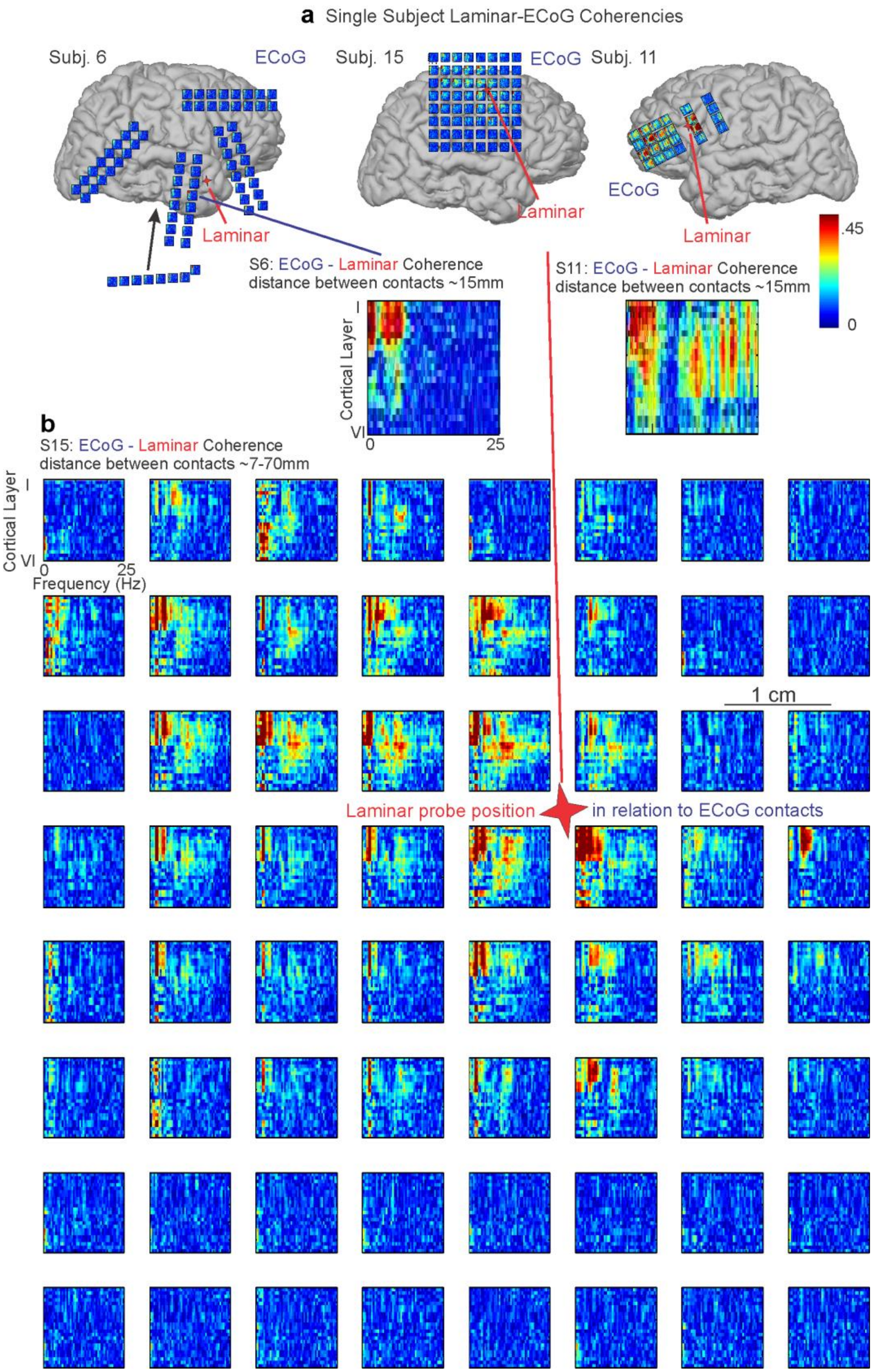

(**a**) Coherence (frequency vs. cortical depth) of individual ECoG contacts with the laminar probe in three individual participants (for Subj. 7 see **Fig. 3**). Slow activity measured by ECoG is consistently coherent with supragranular LFPg measured by the laminar array. (**b**) Each panel displays the coherence between a single ECoG contact in S15 and the 23 channels of the laminar probe, from 1 to 25 Hz. All 64 ECoG channels are shown.

Supplementary Fig. S7

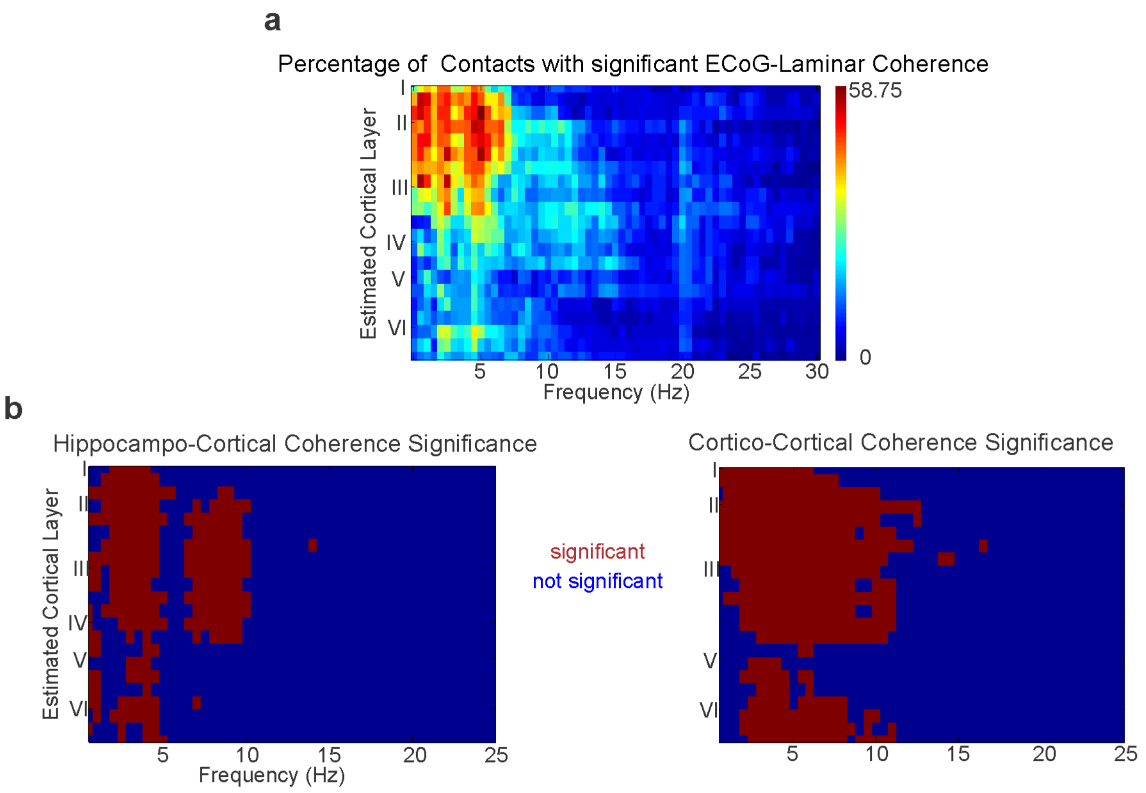

Statistical significance of ECoG-Laminar coherencies. (**a**) The average percentage of ECoG contacts which are significantly coherent with each laminar contact in each frequency band ($p < .05$, Bonferroni Corrected), averaged across 4 subjects. Note that superficial slow activity in superficial layers is coherent with the greatest percentage of ECoG contacts (out of the closest 20 to the laminar probe). (**b**) The significance of coherence between a laminar probe and macroelectrode recordings within the hippocampus and the cortex (n=1). Hippocampo-Cortical and Cortico-Cortical coherence are significant within similar layers and frequencies.

Supplementary Fig. S8

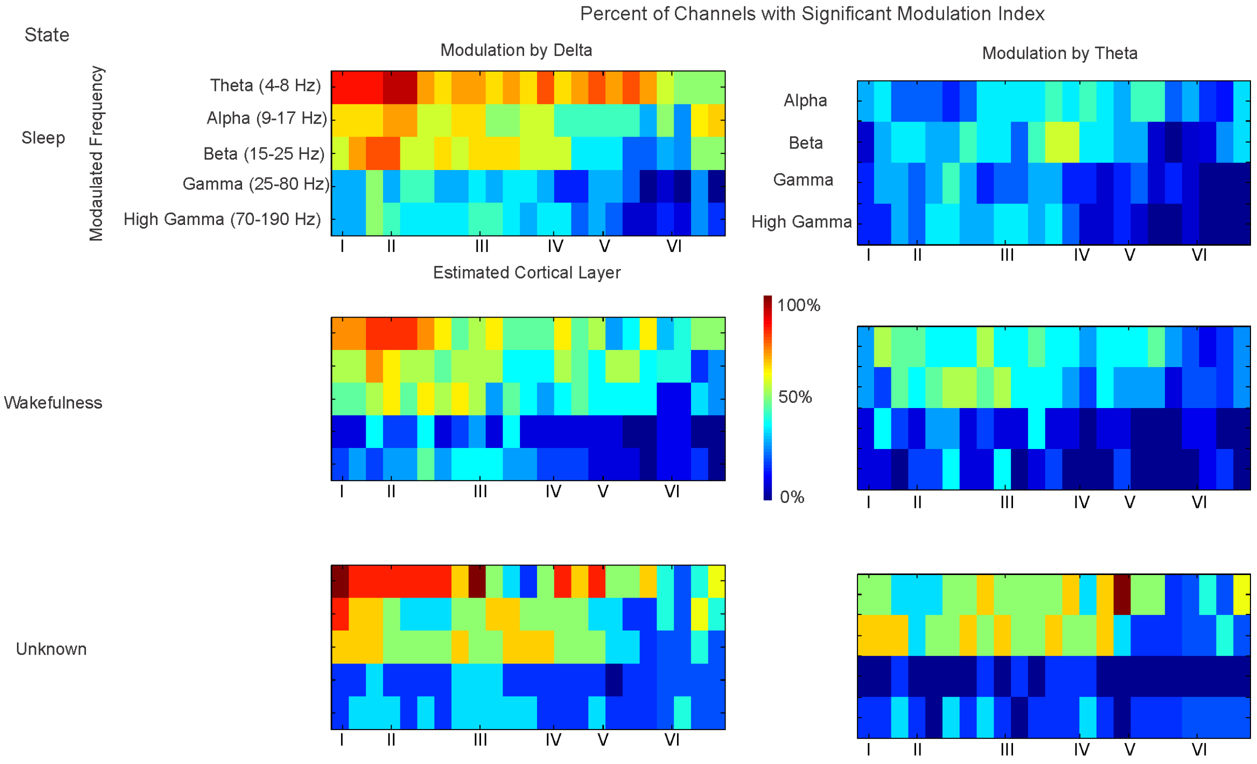

Summary statistical significance of phase-amplitude coupling analysis. The percentage of subjects with significant PAC at each modulating/modulated frequency pair and channel within each behavioral state (see **Supplementary Fig. S3**). Note that although the proportion of subjects with significant PAC for a given channel was sometimes low, the percentage of subjects in each state that had at least one channel with significant PAC ranged from 45 – 100 %.

Supplementary Fig. S9

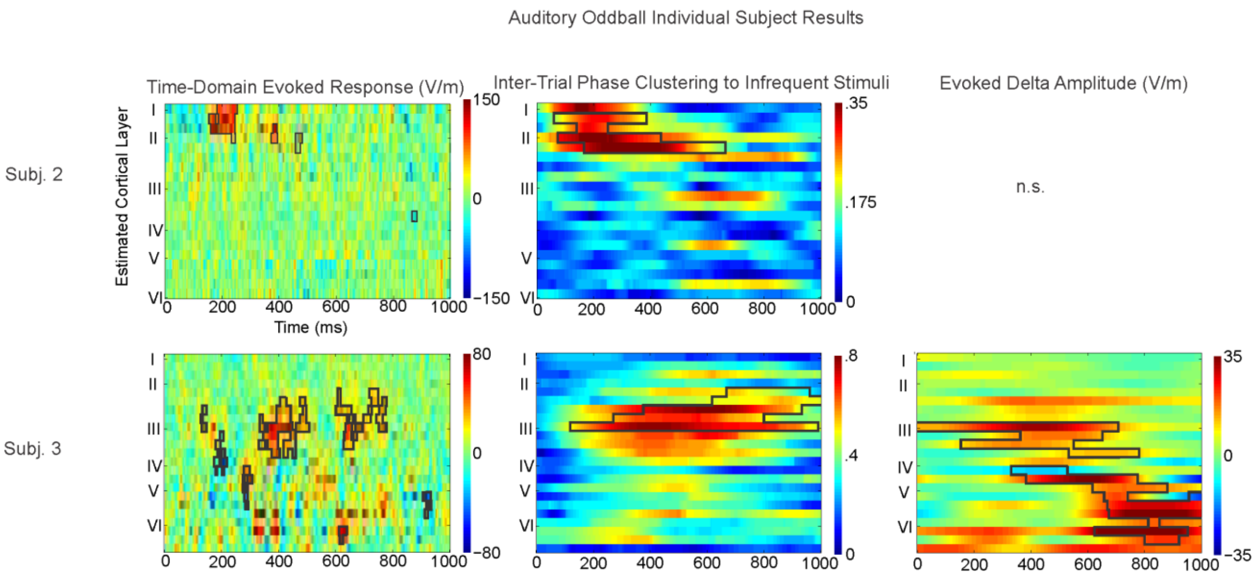

Single subject results and statistical significance for the auditory oddball paradigm. As in **Fig. 5 c-d**, black lines limn clusters which are significantly different between conditions (Nonparametric cluster test, p<.01). The evoked response (time-domain infrequent-frequent average), ITPC values for infrequent targets, and delta-power differences between conditions are plotted for Subjects 2-3 (see **Fig. 5** for Subject 1). Note that delta-power is not plotted for Subject 2, as no significant differences were observed**.**
